# Correction to: Al-induced proteomics changes in tomato plants over-expressing a glyoxalase I gene

**DOI:** 10.1093/hr/uhad060

**Published:** 2023-01-19

This is a correction to: Xiudong Sun, Hui Li, Santosh Thapa, Sasikiran Reddy Sangireddy, Xiaobo Pei, Wei Liu, Yuping Jiang, Shaolan Yang, Dafeng Hui, Sarabjit Bhatti, Suping Zhou, Yong Yang, Tara Fish, Theodore W Thannhauser, Al-induced proteomics changes in tomato plants over-expressing a glyoxalase I gene, Horticulture Research, Volume 7, 2020, 43, https://doi.org/10.1038/s41438-020-0264-x.

In the originally published version of this manuscript, Xiudong Sun’s name was misspelled as Xudong Sun.

One of Xiudong Sun’s affiliations was incorrectly written as College of Horticulture, Shandong Agricultural University, Taian, Shandong, P.R. China. The correct affiliation is College of Horticulture Science and Engineering, Shandong Agricultural University, Taian, Shandong, P.R. China.

These details have been corrected only in this correction notice to preserve the published version of record.

